# Knowledge of multidrug-resistant tuberculosis amongst Durban University of Technology students in KwaZulu-Natal, South Africa: the need for integrating public health education

**DOI:** 10.4314/ahs.v22i2.21

**Published:** 2022-06

**Authors:** Poovendhree Reddy, Udhavana Ramraj, Lauren Chetty

**Affiliations:** Department of Community Health Studies, Faculty of Health Sciences, Durban University of Technology, Durban, South Africa, 4000

**Keywords:** Multi drug resistant TB, health education, public health, risk factors

## Abstract

**Background:**

Kwazulu-Natal is the epicenter of South Africa's Multidrug-resistant Tuberculosis (MDR-TB) burden which represents a growing threat to public health. Knowledge and awareness of MDR-TB are crucial for effective management and University students are an important vehicle for knowledge transfer of public health education.

**Objective:**

This study aimed to evaluate the knowledge of MDR TB and risk factors for transmission, prevention, treatment and control of MDR-TB among Durban University of Technology (DUT) students.

**Methods:**

This quantitative cross-sectional study was conducted among 150 randomly sampled undergraduate students from 3 faculties and data was collected using a validated questionnaire.

**Results:**

While a majority of participants (70.67%) had previous knowledge on TB, only 30.67% knew of MDR-TB. Only 23.49% of students reported knowledge of preventative measures associated with MDR TB. Women had a lower probability of having knowledge of MDR-TB compared to men (OR=0.45; CI:0.22,0.95; p<0.05) and students from the Accounting and Informatics faculty were less likely to believe that MDR-TB was a life-threatening illness (OR=0.24; CI:0.05,1.44; p<0.05) and showed limited knowledge of MDR-TB transmission.

**Conclusion:**

This study showed that students lacked knowledge of MDR-TB with respect to risk factors, treatment and prevention, which necessitates intervention strategies at a tertiary level to educate and inform students about MDR-TB.

## Introduction

The COVID-19 pandemic has taught us several invaluable public health lessons. Uncontrolled infection and disease, particularly when the mode of transmission is respiratory, has the ability to bring the global environment to its proverbial knees. Infection control practices have been taught repeatedly in epidemiology and medical curricula to a select few healthcare professionals, but we argue that health education related to communicable diseases, in particular, should be rolled out at every possible opportunity, particularly to young people, so that they take it forward in their personal lives, their extended family environment and their future workplaces. In South Africa (SA), tuberculosis (TB) and HIV/AIDS is part of the quadruple burden of disease which is a cocktail of four colliding epidemics and South Africans are becoming infected with TB at a rate rivalled only by India and China^1^. It is particularly relevant in the Kwazulu-Natal context, which is the epicenter of the HIV/AIDS and TB co-infection epidemic and has been associated with high rates of drug resistant TB (MDR TB)^2^.

MDR-TB is resistant to both rifampicin and isoniazid which are the conventional first line treatment for TB 3. It had been estimated that 9.6% of all TB cases in South Africa are MDR-TB, thereby ranking South Africa as one of the highest MDR-TB burdened countries in the world^4^,^5^. Most cases of MDR-TB were detected in the provinces of KwaZulu-Natal, Western Cape and Eastern Cape^6^. MDR-TB poses a major threat to public health control programs as treatment is less effective, more complex and costly compared to drug-susceptible disease^7^,^8^. The development of MDR-TB could be attributed to several factors, including poor adherence to treatment, inadequate clinical management, drug malabsorption and unstable drug supply^9^. Risk factors associated with the increased incidence of MDR-TB include HIV^10^, ^11^, ^12^, ^13^; overcrowding^14^ and the lack of knowledge regarding diagnosis, treatment, prevention and control^10^, ^15^, ^2^, ^16^, ^17^.

Health literacy is touted to accommodate the three domains of health: healthcare, disease prevention, and health promotion. Health literacy is important for improving prevention and control of infectious diseases, whereas health knowledge and behavior are important components of health literacy^18^. Health education, which is a component of public health education may slow spread of infectious diseases such as MDR-TB. Health providers' knowledge of TB symptoms, transmission and diagnosis is higher in South Africa than in most other high burden countries^19^, ^20^. TB knowledge in the general population is however not adequately understood. The South African National Strategic Plan for HIV, STI and TB advocates that understanding the population's TB knowledge and practices is the first step towards preventing and managing the disease. The Department of Basic Education (DBE) in SA has recognised that TB is not just a health challenge, but also impacts on our education, economic, social and cultural systems. They released an Integrated Strategy on HIV, STIs and TB in 2012 to intensify efforts at the school level in the prevention, diagnosis, treatment, care and support for these diseases. Materials available to learners and teachers included information on MDR-TB and are still available online to students and educators to date^21^.

The National Policy on HIV, Sexually Transmitted Infections and Tuberculosis was developed by the DBE in 2017 to ensure prevention of disease and promotion of health and well-being among both learner and teachers. One of the goals of the policy was to ensure “increased knowledge, cognitive skills and information about safer sex, life skills in general and HIV, STIs and TB in particular, to inform the life choices of all learners, educators, school support staff and officials and protect them from infection and disease”^22^. Based on these initiatives at the basic education level, we anticipated that undergraduate students would have some knowledge of transmission, symptoms, treatment and risk factors associated with TB.

Most studies documenting knowledge, attitudes and practices related to TB among University populations in South Africa and internationally have been related to undergraduate medical or health sciences students^23^, ^24^, ^25^, ^16^. Adequate knowledge of TB epidemiology and control is critically important for this population as they can be exposed to TB infection during clinic rotations while training^16^ and they are part of the next generation healthcare team against such infectious diseases. However, there is limited data for the general university population in the South African context and it is this group of students that has the potential to pay it forward; to their families, friends and communities. It is clear that educational institutions need to share the responsibility for creating opportunities for best practices with respect to health promotion. Given that the Durban University of Technology (DUT) is a point of entry for students from various regions in KZN, representing different socioeconomic strata, it is important to ensure that students exiting have a social responsibility that is embodied by an increased awareness and knowledge of the health issues threatening both lives and livelihoods. Educational investment is wasted if it is not accompanied by knowledge of healthy living and disease prevention. This study aimed to evaluate the knowledge of MDR TB and risk factors for the transmission, prevention, treatment and control of MDR-TB among DUT students with the intention of recommending an educational intervention among tertiary level students.

## Methodology

### Study design and site

A quantitative cross-sectional study was conducted at the Durban University of Technology (DUT) in KwaZulu-Natal. DUT comprises of five different campuses which include City campus, ML Sultan, Indumiso Campus, Steve Biko and Ritson Campus. Three faculties; Engineering and the Built Environment (EBE), Applied Sciences (AS) and Accounting and Informatics (AI) situated at the Ritson and Steve Biko campuses were targeted as these had the largest number of students.

Ethical approval was obtained from the DUT Institutional Research and Ethics Committee (BE042/18). Gatekeepers permission was sought from the institution, while informed consent was granted by all study participants.

### Participant sampling

Stratified random sampling was used to ensure that all 3 faculties were adequately represented. Only undergraduate students registered at DUT for the 2019 period in their final year of study were included in the sample (N=150). We assumed that the students from the chosen faculties would have received very limited health education as part of their core curriculum. In addition, final year students were chosen as they should have acquired some medical information, albeit from sources other than curriculum, during their university experience.

### Data collection

A validated questionnaire adapted from Behnaz^25^ and Elmi^26^ was used for data collection. The questionnaire was self-administered and comprised of questions relating to demographic characteristics, general knowledge of MDR-TB, knowledge of the risk factors for transmission, prevention, treatment and control of MDR-TB. The questionnaire was piloted prior to use to improve validity and reliability.

### Data analysis

Data was analyzed using STATA version 12 (Statacorp). Descriptive statistics included frequency counts, percentages for categorical variables and mean and standard deviation for age. Data was divided into questions relating to clinical knowledge of MDR-TB and knowledge of transmission of MDR-TB. The chi squared test (χ2) was used to assess participants' responses by sex and faculty. Logistic regression modelling, which included variables showing statistical significance with bivariate testing, was used to examine factors associated with knowledge of MDR-TB among the respondents. A p-value ≤ 0.05 was considered statistically significant at a 95% CI.

## Results

A total of 150 final year undergraduate students from the faculties of Engineering for the Built Environment, Applied Sciences and Accounting and Informatics participated in this study. There were more males than females (83 vs 64) with a mean age of 21.81(2.09) years (Table 1). The African race was predominant (105, 70%) and all three faculties were equitably represented. Table 2 presents clinical knowledge of MDR-TB among the university students stratified by gender and faculty. While most students reported knowledge of TB (70.6%), only 30.7% reported knowledge of MDR-TB. Males were better informed about MDR-TB compared to females (p<0.005). Approximately, 28% of EBE students, 33% of AS students and 26% of AI students did not have previous knowledge of TB and very few had knowledge of MDRTB in each faculty.

**Table 1:** Demographic characteristics of participating students (N=150)

Demographics	n (%)
**Gender**	
Male	83(55.33)
Female	64(42.67)
**Age (mean, SD)**	21.81(2.09)
**Race**	
African	105(70)
Colored	10(6.67)
Indian	33(22)
**Faculty**	
Engineering for the Built Environment (EBE)	54(36.24)
Applied Sciences (AS)	49(32.89)
Accounting and Informatics (AI)	46(30.87)

**Table 2:** Clinical knowledge of MDR-TB among university students (N=150)

		Gender n (%)		Faculty		
	
Knowledge of TB	Total n(%)	Male	Female	EBE	AS	AI
*Students had knowledge of TB*						
Yes	106(70.67)	56(65.88)	50(76.92)	39(72.22)	33(67.35)	34(73.91)
No	44(29.33)	29(34.12)	15(23.08)	15(27.78)	16(32.65)	12(26.09)
*Students had knowledge of MDR-TB*						
Yes	46(30.67)*	32(37.65)	14(21.54)	17(31.48)	17(34.69)	12(26.09)
No	104(69.33)	53(62.35)	51(78.46)	37(68.52)	32(65.31)	34(73.91)
*Students had knowledge of symptoms of MDR-TB*						
Coughing that lasts 3 or more weeks						
Yes	28(18.67)*	22(25.88)	6(9.23)	10(18.52)	8(16.33)	10(21.74)
No	122(81.33)	63(74.12)	59(90.77)	44(81.48)	41(83.67)	36(78.26)
Coughing up blood						
Yes	42(28)	28(32.94)	14(21.54)	11(20.37)	14(28.57)	16(34.78)
No	108(72)	57(67.06)	51(78.46)	43(79.63)	35(71.43)	30(65.22)
Chest pain						
Yes	31(20.67)	19(22.35)	12(18.46)	11(20.37)	11(22.45)	9(20.81)
No	119(79.33)	66(77.65)	53(81.54)	43(79.63)	38(77.55)	37(80.43)
Weight loss						
Yes	29(19.33)	18(21.18)	11(16.92)	9(16.67)	9(18.37)	11(23.91)
No	121(80.67)	67(78.82)	54(83.08)	45(83.33)	40(81.63)	35(76.09)
Fever						
Yes	32(21.48)	16(19.05)	16(24.62)	9(16.67)	14(28.57)	9(20)
No	117(78.52)	68(80.95)	49(75.38)	45(83.33)	35(71.43)	36(80)
Night sweats						
Yes	30(20)	16(18.82)	14(21.54)	11(20.37)	7(14.29)	11(23.91)
No	120(80)	69(81.18)	51(78.46)	43(79.63)	42(85.71)	35(76.09)
All of the above						
Yes	87(58)	46(54.12)	41(63.08)	34(62.96)	28(57.14)	25(54.35)
No	63(42)	39(45.88)	24(36.92)	20(37.04)	21(42.86)	21(45.65)
*MDR-TB is a life threatening illness*						
Yes	98(65.33)	55(64.71)	43(66.15)	39(72.22)*	32(65.31)	26(56.52)
No	28(18.67)	18(21.18)	10(15.38)	6(11.11)	17(34.69)	5(10.87)
Don't know	24(16)	12(14.12)	12(18.46)	9(16.67)	0	15(32.61)
*MDR-TB only affects the lungs*						
Yes	49(32.67)*	33(38.82)	16(24.62)	16(29.63)	16(32.65)	17(36.96)
No	46(30.67)	28(32.94)	18(27.69)	15(27.78)	19(38.78)	12(26.09)
Don't know	55(36.67)	24(28.24)	31(47.69)	23(42.59)	14(28.57)	17(36.96)
*People who do not take their TB medication as* *required can be infected with MDR-TB*						
Yes	107(71.33)	57(67.06)	50(76.92)	42(77.78)	35(71.43)	29(63.04)
No	6(4)	5(5.88)	1(1.54)	3(5.56)	2(4.08)	1(2.17)
Don't know	37(24.67)	23(27.06)	14(21.54)	9(16.67)	12(24.49)	16(34.78)
*MDR-TB can be treated*						
Yes	82(54.67)	47(55.29)	35(53.85)	29(53.70)	26(53.06)	27(58.70)
No	26(17.33)	17(20)	9(13.85)	8(14.81)	11(22.45)	6(13.04)
Don't know	41(27.33)	21(24.71)	20(30.77)	17(31.48)	11(22.45)	13(28.26)
*Duration of treatment*						
6–12 months	29(23.39)	13(19.12)	16(28.57)	14(30.43)	9(23.68)	6(15)
12 – 18 months	29(23.39)	16(23.53)	13(23.21)	8(17.39)	13(34.21)	8(20)
More than 18 months	24(19.35)	18(26.47)	6(10.71)	7(15.22)	4(10.53)	13(32.50)
*Students knew when an infected person should* *stop MDR-TB medication*						
A person should stop taking their medication once they started feeling better	14(9.40)	10(11.76)	4(6.25)	2(3.70)	6(12.50)	6(13.04)
A person should stop taking their medication as prescribed by the doctor	131(87.92)	72(84.71)	59(92.19)	49(90.74)	41(85.42)	40(86.96)
Don't know	4(2.68)	3(3.53)	1(1.56)	3(5.56)	1(2.08)	0
*MDR-TB can be prevented by BCG vaccine*						
Yes	31(20.81)	21(25)	10(15.38)	12(22.22)	9(18.75)	10(21.74)
No	25(16.78)	14(16.67)	49(58.33)	4(7.41)	12(25)	8(17.39)
Don't know	93(62.42)	49(58.33)	44(67.69)	38(70.37)	27(56.25)	28(60.87)
*Students had knowledge of preventative measures*						
Yes	35(23.49)	20(23.81)	15(23.08)	12(22.64)	13(26.53)	10(21.74)
No	114(76.51)	64(76.19)	50(76.92)	41(77.36)	36(73.47)	36(78.26)

* p<0.005

With respect to knowledge of symptoms, only 18,67% of students identified coughing that lasts 3 or more weeks to be one of the symptoms of MDR-TB, and it was reported by more men than women (p<0.005). More male students recognized that coughing up blood (32.94%), chest pains (22.35%), weight loss (21.18%), fever (19.05%) and night sweats (18.82%) were all symptoms of MDR-TB compared to females. There were no significant differences between the 3 faculties with respect to the knowledge of symptoms. Only two thirds of the sample identified MDR-TB as a life-threatening illness. Men were more knowledgeable about MDR-TB only affecting the lungs compared to women (p<0.005), while almost 66% of the population either disagreed or were unsure. A total of 67 participants (42.66%) felt that MDR-TB could not be treated or were unsure. There were relatively fewer students (n = 82) who knew the exact duration of treatment, with 29 (23.39%) choosing the option that treatment is between 6 to 12 months, 29 (23.39) chose between 12 to 18 months, while 24 (19.35) students believed it to be more than 18 months of treatment.

Knowledge of MDR-TB transmission among university students shown in Table 3 is stratified by gender and faculty. Almost 57% of all students agreed that MDR-TB was contagious, however, 36.67% were “not sure”. There were statistically significant differences related to knowledge of the modes of transmission among the 3 faculties, however, it was disconcerting to note that almost 20% of students had no knowledge of transmission. Approximately 27.52% of students felt that drug addicts or abusers were most at risk of contracting MDR-TB (p<0.005), however, 18.79% students had no knowledge of risk. Logistic regression analysis showed that women had a lower probability of having knowledge of MDR-TB compared to men (OR=0.45; CI:0.22,0.95; p<0.05). In terms of faculty, AS students less likely than EBE and AI students to believe that MDR-TB was a life-threatening illness (OR=0.24; CI:0.05,1.44; p<0.05). Similarly, AI was less likely to know about airborne transmission compared to other faculties; indeed students from AI had very poor knowledge of MDR-TB transmission.

**Table 3:** Knowledge of MDR-TB transmission among university students (N=150)

		Gender n (%)		Faculty		
	
	Total n(%)	Male	Female	EBE	AS	AI
*MDR-TB is contagious*						
Yes	85(56.67)	53(62.35)	32(49.23)	30(55.56)	30(61.22)	25(54.35)
No	10(6.67)	6(7.06)	4(6.15)	2(3.70)	6(12.24)	2(4.35)
Don't know	55(36.67)	26(30.59)	29(44.62)	22(40.74)	13(26.53)	19(41.30)
*Transmission of MDR-TB*						
Transmitted through the air when an infected person coughs, sneezes or speaks	111(74.50)	60(71.43)	51(78.46)	45(83.33)*	38(79.17)	28(60.87)
Passed by sharing the same utensils as an infected person	8(5.37)	7(8.33)	1(1.54)	1(1.85)*	4(8.33)	3(6.52)
No knowledge of transmission	30(20.13)	17(20.24)	13(20)	8(14.81)*	6(12.50)	15(32.61)
*Students' belief of people at risk for* *MDR-TB*						
People infected with HIV	51(34)	29(34.12)	22(33.85)	17(31.48)	20(40.82)	13(28.26)
People with diabetes	12(8)	8(9.41)	4(6.15)	2(3.70)	6(12.24)	4(8.70)
Drug addicts/ abusers	41(27.33)	21(24.71)	20(30.77)	9(16.67)*	21(42.86)	11(23.91)
People living in overcrowded areas	58(38.67)	32(37.65)	26(40)	17(31.48)	21(42.86)	19(41.30)
All of the above	41(27.33)	24(28.24)	17(26.15)	18(33.33)	11(22.45)	12(26.09)
No knowledge of risk	28(18.67)	18(21.18)	10(15.38)	12(22.22)*	4(8.16)	12(26.09)
*Health care workers must wear masks* *when treating patients with MDR-TB*						
Yes	124(82.67)	65(76.47)	59(90.77)	48(88.89)	37(75.51)	38(82.61)
No	10(6.67)	7(8.24)	3(4.62)	2(3.70)	6(12.24)	2(4.35)
Don't know	16(10.67)	13(15.29)	3(4.62)	4(7.41)	6(12.24)	6(13.04)

* p<0.005

## Discussion

This study aimed to evaluate the knowledge of MDR TB and risk factors for the transmission, prevention, treatment and control of MDR-TB among DUT students. University students are an important vehicle for knowledge transfer of public health education to both society and the workforce. Knowledge and awareness of MDRTB are crucial for effective treatment, prevention and control of this communicable disease. While a majority of participants (70.67%) had previous knowledge on TB, only 30.67% knew of MDR-TB. In spite of the well-recognized HIV-TB co-morbidity in South Africa (with Kwazulu-Natal as the epicenter), only a 25% of students acknowledged that HIV patients were at greater risk of acquiring TB, whilst 92% did not know of any MDR TB drugs. Only 23.49% of students reported knowledge of preventative measures associated with MDR TB. Our study showed that students lacked the necessary knowledge of MDR-TB with respect to risk factors, treatment and prevention.

The findings from this study show that a majority (70.67%) of participants had previous knowledge on TB (Table 1), however only 30.67% knew of MDR-TB, which was similar to a study conducted in Pakistan where only 36.4% of students surveyed knew about MDR-TB^27^. This is concerning as both Pakistan and South Africa are two of the highest TB burdened countries in the world and one of the contributing factors to the incidence rates may be a lack knowledge on important aspects of this disease. However, it was noted that more than half (58%) of participants recognized that all of the symptoms listed in the questionnaire were that of MDR-TB. This result differed from a study in China which concluded that less than half of the students who had participated had recognized the symptoms^16^. Although the majority of the participants (56.67%) had known that MDR-TB is contagious (57%) and is a life-threatening disease (65%), 37% did not know whether MDR-TB affects the lungs only or not. It is more likely that students may have a generalized perception of the disease which is based on common sense, rather than specific knowledge as they were able to answer general questions related to transmission and pathology, but struggled with detailed questions. With regards to the transmission of the disease, this study showed that 74.5% of participants knew that MDR-TB may be transmitted through coughing or sneezing. In contrast with this, other studies have reported that only one third of respondents provide correct answers when asked about transmission of the disease^28^, which indicated that knowledge of drug susceptible TB was good among students.

Only 18% of participants identified all factors pertaining to increased risk for MDR TB, while 25% perceived it to be overcrowding and 22% perceived it to be a positive HIV status. It was evident that there is stigma associated with the at-risk population for MDR TB, particularly as it is associated with the HIV/AIDS epidemic. More than half of the participants (54.67%) knew that MDR-TB is a treatable disease. Elmi^26^ have asserted that if more people understood that MDR-TB is a treatable disease, it would increase the number of people seeking early treatment which may decrease disease incidence in highly burdened countries. A persons' knowledge on the treatment duration of MDR-TB is important as compliance is essential for a successful prognosis; only 19% of respondents knew that MDR-TB is treated for longer than 18 months. Studies describing the knowledge and risk perception of HIV, and TB in relation to HIV, have been conducted among university students in South Africa 27, 28,29. Evans^30^ interviewed students from three universities across South Africa and found that 55.3% (n=438) and 52.1% (n=412) were categorized as having poor TB or HIV knowledge while 43.4% (n=344) and 39.8% (n=315) were categorized as having high TB or HIV risk perception.

Compared with female participants, male students were more likely to have poor knowledge of HIV. This highlighted the need to enhance health promotion activities and amongst university students and provide additional support as the university campus is an ideal opportunity to engage with adolescents.

This study revealed that knowledge among University students of an important public health threat in SA was very limited. There is, therefore, an urgent need for intervention strategies at tertiary level to further educate and inform students about TB and MDR-TB. Currently, HIV education is well represented in local Higher Education Institutions (HEIs). The University of Kwazulu-Natal (UKZN) advocates that all their courses have some component of HIV education in order to raise student awareness. Support is provided to staff to develop and integrate HIV/AIDS issues into theirurricula (UKZN AIDS policy). In addition to integrating HIV education into the curriculum, the DUT has in an HIV center which seeks to transform the DUT community into an HIV/AIDS competent community through effective knowledge and skills. Education on conditions that constitute the quadruple burden of disease in South Africa is important for the mitigation and control of public health disease threats. In countries with a high relatively high MDR-TB burden, it is important that all opportunities to raise population awareness about the disease are used to the optimum. Universities may be an important avenue for MDR-TB health education given the multiplier effect such intervention is likely to have on the students, their families, communities and larger social networks. The University should also consider creating an online platform which could be managed by the University clinics or the Health Science departments which promote MDRTB information as well information on other diseases. This should be freely accessible to students and it may be a more effective method as students are always using their smartphones and other digital devices. This initiative, coupled with ongoing media releases and TB mitigation programs as part of South Africa's public health strategy, has the potential to decrease vulnerability and increase resilience among students and their contacts.

## Figures and Tables

**Table 4:** Logistic regression analysis of factors associated with knowledge of MDR-TB (N=150)

	Gender	Faculty	
	
	Female	AS	AI
	
	OR (95% CI)	OR (95% CI)	OR (95% CI)
Students had knowledge of MDR-TB	0.45(0.22;0.95)*	1.16(0.51;2.63)	0.77(0.32;1.84)
MDR-TB is contagious	1.16(0.31;4.29)	0.28(0.05;1.44)	0.85(0.11;6.26)
MDR-TB is a life-threatening illness	1.48(0.63;3.46)	0.24(0.08;0.66)*	1.03(0.29;3.61)
MDR-TB only affects the lungs	1.28(0.63;2.60)	0.61(0.27;1.39)	1.09(0.45;2.65)
Transmitted through the air when an infected person coughs, sneezes or speaks	1.52(0.71;3.22)	0.69(0.26;1.84)	0.31(0.12;0.79)*
Passed by sharing the same utensils as an infected person	0.17(0.02;1.45)	4.71(0.51;43.68)	3.70(0.37;36.83)
No knowledge of transmission	1(0.45;2.24)	0.80 (0.25;2.50)	2.78 (1.05;7.35)*

Male and EBE were the reference categories

* p-value<0.05 was considered statistically significant
